# Discovery of a Potent Anti-Yeast Triterpenoid Saponin, Clematoside-S from *Urena lobata* L.

**DOI:** 10.3390/ijms16034731

**Published:** 2015-03-02

**Authors:** Xiao-Ling Gao, Ying Liao, Jie Wang, Xiao-Yan Liu, Kai Zhong, Yi-Na Huang, Hong Gao, Bo Gao, Zheng-Jun Xu

**Affiliations:** 1 Rice Research Institute, Sichuan Agricultural University, Wenjiang 611130, China; E-Mail: gaoxl0908@hotmail.com; 2 College of Life Science, Sichuan Normal University, Chengdu 610101, China; E-Mail: ying_liao@msn.com; 3 College of Light Industry, Textile and Food Engineering, Sichuan University, Chengdu 610065, China; E-Mails: wjie0207@163.com (J.W.); lxy19831231@126.com (X.-Y.L.); eric211@163.com (K.Z.); gao523@hotmail.com (H.G.); 4 West China School of Public Health, Sichuan University, Chengdu 610041, China; E-Mail: dir0932@sina.com

**Keywords:** *Urena lobata*, triterpene saponin, clematoside-S, anti-yeast activity

## Abstract

*Urena lobata* has been used as a traditional medicinal plant in India and China. In this study, we investigated the antimicrobial activity and isolated the active compound from the leaves of *U*. *lobata*. The 80% ethanol extract from *U*. *lobata* leaves showed an effective anti-yeast activity against *Saccharomyces cerevisiae* (*S*. *cerevisiae*) strains. Using a combination of chromatographic methods, (−)-trachelogenin (**1**) and clematoside-S (**2**) were isolated from this plant for the first time, and their chemical structure was identified by mass spectrometry (MS) and extensive nuclear magnetic resonance (NMR) data analysis. In addition, **1** was found to be inactive against all of the test microorganisms in the antimicrobial assay, whereas **2** exhibits a specific anti-yeast activity against *S*. *cerevisiae* strains with diameter of inhibition zones in the range from 11 to 20 mm. Furthermore, the MIC (minimum inhibitory concentration) and MBC (minimum bactericidal concentration) values of **2** against *S*. *cerevisiae* strains were detected to be in the ranges of 0.61 to 9.8 μg/mL and 2.42 to 9.8 μg/mL, respectively. This is the first report of **2** with a specific anti-yeast activity. The above result suggests the potential application of *U*. *lobata* to be used as a natural anti-yeast agent in food preservation.

## 1. Introduction

Food spoilage by the food-related yeast causes the deterioration of a wide range of foodstuffs such as wines, milk, fruit and vegetable juices, soft drink or meat. Spoilage yeasts not only significantly influence the cost and availability of foods and beverages, but also lead to economic losses in food industry [1,2,3]. Notably, *Saccharomyces cerevisiae* is listed on one of the most significant spoilage yeasts for fruit juices and soft drinks [4]. Although chemical preservatives can exclude yeast spoilage, there is a strong consumer demand to avoid or diminish the use of artificial preservatives. Therefore, much effort has been expended in the search for effective natural compounds from herbs and spices in order to control food spoilage caused by yeast and replace existing synthetic antibiotics in foodstuffs [5,6].

**Figure 1:**
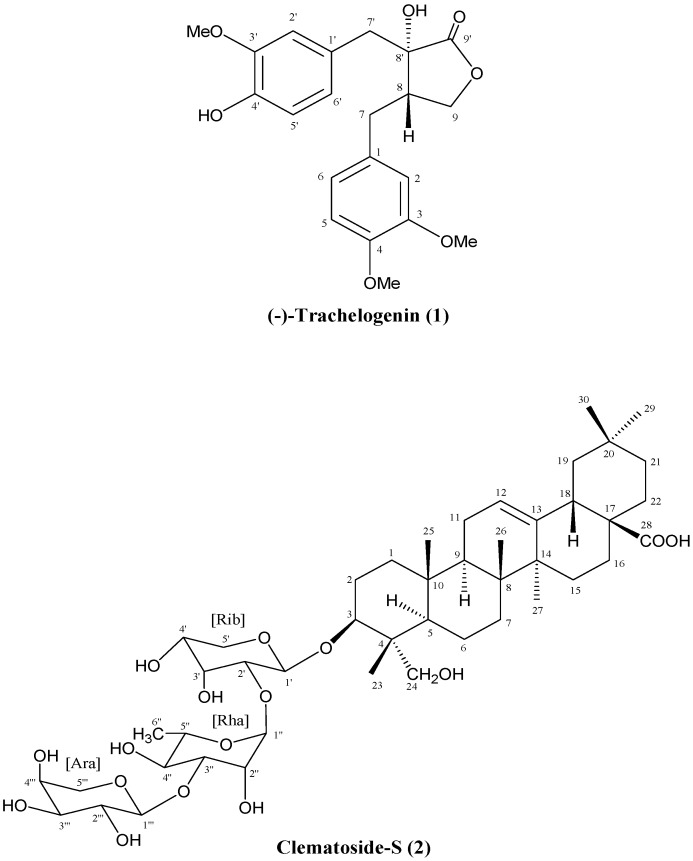
Chemical structure of (−)-trachelogenin (**1**) and clematoside-S (**2**).

*Urena lobata* L., indigenous in China, is a number of the Malvaceae family. The plant, commonly known as Ye-Mian-Hua in China, is a popular folk medicine as diuretic, febrifuge, and also as a remedy for dysentery, cough, dropsy and rheumatism to exhibit a variety of biological activities, including antioxidant, anti-inflammatory, anti-proliferative, and antibacterial activities [7,8,9]. Recently, it has been reported that the methanolic extract from *U*. *lobata* leaves showed antibacterial activity against *Micrococcus roseus* and *Mycobacterium smegmatis* [10]. Some phytochemical compounds such as flavonoids, triglycerides and lignans have been isolated from this plant [11,12,13]. In the course of our ongoing program on identifying antimicrobial principles from natural materials, we found that the aqueous ethanolic extract from the leaves of *U*. *lobata* showed significant anti-yeast activity using *S*. *cerevisiae* as an indicator. In the present study, we have attempted to isolate the anti-yeast substance(s) from the leaves of *U*. *lobata*. On bioassay guided fractionation of aqueous ethanolic extract, further work led to the isolation of (−)-trachelogenin (**1**) and clematoside-S (**2**) (Figure 1). Herein, we describe the isolation and structural elucidation of compounds **1**–**2**, together with evaluating their antibacterial and antifungal activities.

## 2. Results and Discussion

### 2.1. Isolation of Compounds **1**–**2** from the Leaves of Urena lobata

**Scheme 1:**
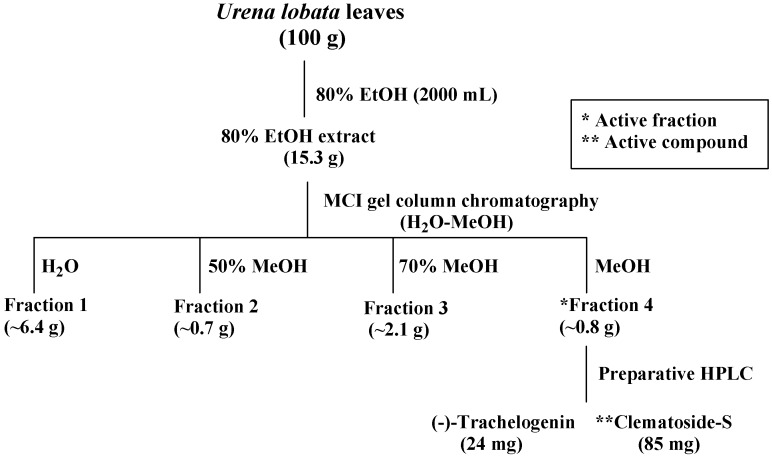
Isolation scheme of (−)-trachelogenin (**1**) and clematoside-S (**2**) from *U*. *lobata*.

Scheme 1 shows the extract and isolation scheme of compounds **1**–**2** from the leaves of *U*. *lobata*. Powdered leaves of *U*. *lobata* were extracted with 80% ethanol and further fractionation was performed with a guidance of inhibitory zone diameter against *S.* *cerevisiae* (ATCC 204508). At a concentration of 10 mg/mL, the crude extract exhibited 15 mm of inhibitory zone diameter against *S.* *cerevisiae*. The extract was subjected to MCI gel column chromatography to give four fractions with a step gradient elution of water-methanol. The active fraction 4 (16 mm of inhibitory zone diameter against *S.* *cerevisiae* at a concentration of 10 mg/mL) eluted with 100% methanol, was further purified by preparative HPLC to afford compounds **1** and **2**. This is the first report of compounds **1**–**2** isolated from *U*. *lobata*.

### 2.2. Identification of Isolated Compounds **1**–**2**

The chemical structures of the isolated compounds **1** and **2** were identified by spectroscopic analyses consisting of MS, ^1^H-NMR, ^13^C-NMR and 2D-NMR data analyses. By comparison with literature data [14,15,16], compound **1** was identified as (−)-trachelogenin: pale-yellow gum; electron spray ionization-mass spectrometry (ESI-MS) (negative), *m*/*z* 387.25 [M − H]^−^; electron spray ionization-high resolution mass spectrometry (ESI-HRMS) *m*/*z* 411.1420 (calcd. for C_21_H_24_O_7_Na, 411.1420); [α]_D_^25^ −32.2° (*c* 0.43, CHCI_3_); ^1^H-NMR δ (methanol-*d*_4_) ppm (*J* in Hz): 2.45 (1H, m, H-8), 2.50 (1H, dd, *J* = 13.6, 4.1, H_a_-7), 2.80 (1H, dd, *J* = 13.6, 5.0, H_b_-7), 2.85 (1H, d, *J* = 13.6, H_a_-7'), 3.12 (1H, d, *J* = 13.6, H_b_-7'), 3.78 (3H, s, 3'-OMe), 3.79 (6H, s, 3 and 4-OMe), 3.97 (1H, d, *J* = 8.8, H_a_-9), 3.98 (1H, d, *J* = 8.8, H_b_-9), 6.57 (1H, dd, *J* = 8.1, 1.9, H-6'), 6.70 (4H, m, H-2, 2', 5' and 6), 6.85 (1H, d, *J* = 8.1, H-5). ^13^C-NMR δ (150 MHz, methanol-*d*_4_) ppm: 30.82 (C-7), 40.50 (C-7'), 43.14 (C-8), 55.04 (3- and 4-OMe), 55.17 (3'-OMe), 70.40 (C-9), 76.01 (C-8'), 111.86 (C-5), 112.53 (C-2), 113.59 (C-2'), 114.69 (C-5'), 120.81 (C-6), 122.68 (C-6'), 126.82 (C-1'), 132.05 (C-1), 145.34 (C-4'), 147.46 (C-3'), 147.76 (C-4), 149.18 (C-3), 179.17 (C-9').

Compound **2** was obtained as white powder. In the FAB-MS spectra (negative mode), deprotonated molecular ion peak of **2** was observed at *m*/*z* 881 [M − H]^−^, while positive fast atom bombardment-mass spectrometry (FAB-MS) showed a highest and base ion peak at *m*/*z* 883 [M + H]^+^. So, these data show that the molecular weight of the active principle is 882 Da. The linked-scan spectrum in negative mode showed the fragmentations from the quasi molecular ion of *m*/*z* 881 [M − H]^−^ to *m*/*z* 750 [M + H − 131]^−^, 732 [M − H − 149]^−^, 604 [M − H − 277]^−^, 585 [M − H − 295]^−^, and 471 [M − H − 410]^−^, which suggests the active compound is a glycoside containing a sugar moiety of the sequence of pentose-deoxyhexose-pentose. The molecular weight of the aglycone is deduced to be 472 Da.

The ^1^H-NMR spectrum apparently showed six tertiary and one secondary methyl signals. The ^13^C-NMR spectrum in CD_3_OD showed 47 signals except for those overlapping with CD_3_OD signals, two of which were assigned to those of acetic acid (δ_C_ 29.09 ppm, δ_C_ 175.76 ppm). The polarization transfer (DEPT)-135 and DEPT-90 NMR spectra enclosed one methine carbon at δ_C_ 49.17 ppm among the signals overlapping with those of CD_3_OD, and showed 7 methyl, 13 methylene, 18 methine, 7 quaternary, and one carbonyl carbon signals to be assigned to the active principle. Since the active principle contains a pentose-deoxyhexose-pentose chain, 30 carbon signals comes from an aglycone, which is expected to be a triterpene. The heteronuclear single quantum coherence (HSQC) spectrum showed the signals of six tertiary and one secondary methyl carbons, the latter of which was assigned to a deoxyhexose by heteronuclear multiple-bond correlation (HMBC) spectrum analysis. The assignments of the protons and carbons are discussed in the following sessions and summarized in Table 1.

The HMQC spectrum showed that a carbinol carbon at δ_C_ 82.28 ppm, which was assigned to C-3 of an aglycone, carries a proton at δ_H_ 3.61 (H-3), and these showed long range couplings to the anomeric proton and carbon of a sugar, suggesting the linkage between the aglycone and a sugar chain. The proton at δ_H_ 3.61 ppm (H-3) showed long range couplings to methyl carbon (C-23) at δ_C_ 13.79 ppm and a quaternary carbon (C-4) at δ_C_ 43.95 ppm. The methyl proton on C-23 long-range coupled to C-4 and a primary carbinyl carbon (C-24) at δ_C_ 64.58 ppm in addition to the carbinol carbon, C-3, and a methine carbon (C-5) at δ_C_ 48.15 ppm. The methine proton at δ_H_ 1.26 ppm on C-5 showed coupling to C-6 at δ_C_ 18.80 ppm, C-10 at δ_C_ 37.61 ppm, and C-25 at δ_C_ 16.40 ppm in addition to C-4, C-23, and C-24. The methyl proton on C-25 showed a coupling to a methylene carbon at δ_C_ 39.67 ppm, which was finally assigned to C-1, in addition to two quaternary carbons at δ_C_ 37.61 ppm (C-10) and δ_C_ 49.0 (C-9). The carbinyl proton at δ_H_ 3.61 ppm (H-3) on C-3 showed vicinal couplings to methylene protons at δ_H_ 1.74 ppm (H-2a, *J* = 4.5 Hz) and δ_H_ 1.85 ppm (H-2b, *J* = 12.2 Hz). These two protons are on the carbon at δ_C_ 26.58 ppm (C-2), showed a geminal coupling (*J* = 17.4 Hz), and vicinal couplings to a proton at δ_H_ 1.60 ppm (H-1a) on the carbon at δ_C_ 39.67 ppm (C-1). H-1 shows a geminal coupling to H-1b at δ_H_ 0.98 ppm, which shows vicinal couplings to H-2a and H-2b. Taking all the information mentioned above, a partial structure (ring A) of the aglycone was determined as shown in Figure 1. Of the two olefinic carbon signals at δ_C_ 123.60 ppm and δ_C_ 145.21 ppm which are later assigned to C-12 and C-13, respectively, C-12 carries an olefinic proton at δ_H_ 5.23 ppm (H-12) which showed long-range couplings to carbons at δ_C_ 24.52 ppm (secondary), δ_C_ 49.0 ppm (C-9), and δ_C_ 42.72 ppm (tertiary, C-18). The HMQC spectrum showed that the carbon at δ_C_ 24.52 ppm carries protons at δ_H_1.87 ppm and δ_H_ 1.89 ppm, both of which showed couplings to the olefinic proton (H-12) at δ_H_ 5.23 ppm in the HMBC spectrum. In addition, one or both of the protons appearing at δ_H_ 1.87 ppm and δ_H_ 1.89 ppm showed couplings to both olefinic carbons. These H-H and C-H couplings suggest that the carbon at δ_C_ 24.52 ppm is vicinal to the carbon at δ_C_ 123.60 ppm (C-12) to be assigned to C-11. A long rage coupling from the proton at δ_H_ 1.62 ppm (H-9) on the methine carbon at δ_C_ 49.00 ppm (C-9) to C-11 was observed. To C-9, were observed long range couplings from H-12 at δ_H_ 5.23 ppm and methyl protons at δ_H_ 0.96 ppm (H_3_-25) and δ_H_ 0.80 ppm (H_3_-26). H-9 couples to C-11 in addition to the carbons at δ_C_ 37.61 ppm (C-10), δ_C_ 48.15 ppm (C-5), and δ_C_ 40.50 ppm (C-8). These couplings enclosed the sequence of C-10/C-9/C-11/C-12/C-13, and the attachment of C-25 on C-10. The methyl protons (H_3_-26) shows a long range coupling to a quaternary carbon at δ_C_ 42.96 ppm, which was assigned to C-14, in addition to C-9, C-10, and the methylene carbon at δ_C_ 33.39 ppm assigned to C-8 through HMQC and H^1^-H^1^ COSY spectrum analyses. Based on the above information, the partial structure constructing rings A, B, and C appeared, and hederagenin came out as the candidate for the aglycone. Comparing our NMR data with those in literatures [17,18,19], the aglycone was unambiguously identified to be hederagenin.

The linked scan MS suggests the sequence of pentosyl-deoxyhexosyl-pentosyl chain. The d configurations of the arabinose and ribose and the l configuration of rhamnose were established after hydrolysis of **2** followed by GC analysis [20,21,22]. GC-MS analysis of the trimethylsilylates of *N*-methoximes of the sugars obtained by acid hydrolysis of **2** showed the major peaks at *t*_Rs_ of 15.29, 15.81, and 17.18 min, respectively. The *t*_Rs_ of these peaks were identical to those of standard silylated samples, showing that the sugar components of the active principle were d-arabinose, d-ribose, and l-rhamnose. The HMBC showed that the carbinyl proton (H-3) at δ_H_ 3.61 ppm long-range coupled to an anomeric carbon (C-1') at δ_C_ 104.63 ppm, and the anomeric proton (1'-H) at δ_H_ 4.51 ppm to C-3 of hederagenin at δ_C_ 82.28 ppm. The *J* value between H-1' and H-2' (δ_H_ 3.69 ppm) on the carbon (C-2') at δ_C_ 76.33 ppm was 6.0 Hz, suggesting the axial-equatorial relation of the two protons, and all signals of the protons and the carbons of the pentose directly attached to hedragenin were assigned as shown in Figure 1 and Table 1. The C-2' and C-3' were distinguished by the long-range coupling from H-5a' and H-5b' to the carbon at δ_C_ 74.21 ppm (C-3'). Based on the chemical shifts of all signals and the *J* value (6.0 Hz) between H-1' and H-2', the pentose was determined to be α-d-ribose (Figure 1). Similarly, all the signals of α-l-rhamnose were assigned as shown in Table 1 and the ether linkage from C-2' of ribose to C-1' of rhamnose was determined by the long-range coupling from H-2' of ribose to C-1'' of rhamnose and that from H-1'' of rhamnose to C-2' of ribose. The third sugar, arabinose, was determined to be β-anomer based on the *J* value (4.3 Hz) between H-1''' and H-2''', and all signals of β-arabinose were assigned as shown Table 1. The long-range coupling from H-3'' to C-1''' and that H-1''' to C-3'' showed the ether linkage between C-3'' of rhamnose and C-1''' of arabinose. Finally, the chemical structure of 2 was determined to be β-d-arabinosyl-(1-3)-α-l-rhamnosyl-(1-2)-α-d-ribosyl-(1-3β)-hederagenin, namely clematoside-S.

**Table 1:** The NMR spectroscopic data for clematoside-S (**2**) in MeOH-*d*_4_.

Position	δ_C_	δ_H_ (Mult., *J* in Hz)	HMBC
1	39.67	0.98 (overlap), 1.60 (m)	-
2	26.58	1.74 (m), 1.85 (m)	-
3	82.28	3.61 (m)	C-4, C-23, C-1'
4	43.95	-	-
5	48.15	1.26 (m)	C-4, C-10, C-23, C-25
6	18.80	1.36 (m), 1.51 (m)	-
7	33.39	1.26 (m), 1.63 (m)	-
8	40.50	-	-
9	49.00	1.62 (m)	C-5, C-8, C-10, C-11
10	37.61	-	
11	24.52	1.87 (m), 1.89 (m)	C-12, C-13
12	123.60	5.23 (m)	-
13	145.21	-	-
14	42.96	-	-
15	28.82	1.07 (m), 1.76 (m)	-
16	20.05	1.61 (m), 2.01 (m)	C-28
17	47.63	-	-
18	42.72	2.84 (m)	C-12, C-13, C-17, C-28
19	47.22	1.12 (m), 1.69 (m)	-
20	31.59	-	-
21	34.88	1.20 (m), 1.39 (m)	-
22	33.81	1.53 (m), 1.74 (m)	-
23	13.79	0.70 (s)	C-3, C-4, C-5, C-24
24	43.95	3.37 (m), 3.53 (m)	C-4, C-23
25	16.40	0.96 (s)	C-1, C-9, C-10
26	17.75	0.80 (s)	C-7, C-8, C-9, C-14
27	26.46	1.17 (s)	C-13, C-14, C-15
28	181.88	-	-
29	33.57	0.90 (s)	C-19, C-20, C-21, C-30
30	23.97	0.93 (s)	C-19, C-20, C-21, C-29
1'	104.63	4.51 (d, *J* = 6.0)	C-3, C-5'
2'	76.33	3.69 (m)	C-1', C-3'
3'	74.21	3.69 (m)	C-1', C-2'
4'	69.64	3.75 (dd, *J* = 13.8, 6.6)	-
5'	65.44	3.5 (d, *J* =6.6), 3.83 (d, *J* = 6.6)	C-1', C-3'
1''	101.52	5.21 (d, *J* = 1.6)	C-2', C-2'', C-3'', C-5''
2''	71.73	4.05 (d, *J* = 2.4)	C-3'', C-4''
3''	80.70	3.83 (d, *J* = 13.8)	C-4'', C-1'''
4''	72.96	3.83 (d, *J* = 13.8)	C-3'', C-5''
5''	70.33	3.90 (d, *J* = 7.2)	-
6''	17.99	1.23 (m)	C-5''
1'''	104.18	4.99 (d, *J* = 4.3)	C-3'', C-3'''
2'''	72.60	3.67 (d, *J* = 3.2)	C-1''', C-3'''
3'''	68.66	3.96 (d, *J* = 3.2)	C-1'''
4'''	70.17	3.76 (dd, *J* = 13.2, 6.6)	C-1''', C-4'''
5'''	65.13	3.67 (d, *J* = 13.2), 3.89 (d, *J* = 13.2)	-

### 2.3. Antimicrobial Activity of Isolated Active Compound

The antimicrobial activity of the 80% ethanol extract from *U*. *lobata* leaves was evaluated using Oxford plate method against five strains of food-borne bacteria (*Eschericha coli*, *Salmonella typhimurium*, *Staphylpcocuus aureus*, *Bacillus subtilis*, and *Bacillus laterosporus*), four strains of fungi (*Aspergillus flavus*, *Aspergillus niger*, *Rhizopus oryzae*, and *Pencicillum citrinum*), and six yeast strains (*Candida albicans*, *Saccharomyces cerevisiae* ATCC 204505, *Saccharomyces cerevisiae*\AY529515.1, *Saccharomyces cerevisiae*\AJ746340.1, *Saccharomyces cerevisiae*\JX103178.1, and *Saccharomyces boulardii*\KG254081.1) as shown in Table 2. The solvent (95% methanol) used as the negative control did not show any activity. At a concentration of 10 mg/mL, the crude extract showed no antibacterial activity. Meanwhile, the extract was inactive against three species of fungi (*A*. *flavus*, *R*. *oryzae*, and *P*. *citrinum*) even at 10 mg/mL. Among the test fungi, the most sensitive strain was *A*. *niger* with the diameter of inhibition zone of 9 mm. The extract showed no activity (10 mg/mL) against *C*. *albicans*. It was worth noting that the extract exhibited remarkable anti-yeast activities against *S*. *cerevisiae* ATCC 204505, *S*. *cerevisiae*\AY529515.1, *S*. *cerevisiae*\AJ746340.1, *S*. *cerevisiae*\JX103178.1, and *S*. *boulardii*\KG254081.1 with the diameter of the inhibition zone in the range from 14 to 17 mm. The result suggest that *U*. *lobata* leaves inhibits selectively the growth of some yeast strains. So, we selected out *S*. *cerevisiae* ATCC 204505 as the indicator for detecting the main anti-yeast substance(s) in *U*. *lobata* leaves. Using a combination of chromatographic methods, (−)-trachelogenin (**1**) and clematoside-S (**2**) were isolated from the 80% ethanol extract of *U*. *lobata* leaves. It was found that **1** showed no activity against all of the selected microorganisms in the antimicrobial assay (Table 2). However, **2** showed promising activity against *S*. *cerevisiae* ATCC 204505 as compared to standard anti-yeast reagent, streptomycin. In addition, **2** exhibited a potent inhibitory effect against the five test yeast strains, except for *C*. *albicans*, with diameter of inhibition zones in the range from 11 to 20 mm.

Further study was carried out to investigate the anti-yeast effect of **2** against *S*. *cerevisiae* ATCC 204505, *S*. *cerevisiae*\AY529515.1, *S. cerevisiae*\AJ746340.1, *S*. *cerevisiae*\JX103178.1, and *S*. *boulardii*\KG254081.1 by measuring the minimum inhibitory concentration (MIC) and the minimum bactericidal concentration (MBC). The MICs and MBCs of **2** to the five test yeast strains were shown in Table 3. These results demonstrated that **2** had certain antibacterial and bactericidal property. In general, the MICs of **2** against the test yeasts were in range from 0.61 to 9.80 μg/mL, and MBCs from 2.42 to 9.80 μg/mL, respectively. Associated with the results of disc diameter of inhibition zone in Table 2, it was clearly indicated that **2** showed the strongest activity against *S*. *cerevisiae* ATCC 204505, whereas it showed the moderate activity against the other yeast strains (Table 3). clematoside-S (**2**) isolated from clematotic species has been reported to exhibit cytotoxic activity against several cancer cells [23]. However, compound **2** has never been studied for antimicrobial activity. Our finding is the first report on the isolation of **2** with anti-yeast activity from *U*. *lobata*. Therefore, it may be proposed that *U*. *lobata* can be used as a natural anti-yeast agent to control food spoilage caused by yeast. However, further study is warranted to provide clear evidence for toxicity profile.

**Table 2:** Antimicrobial activity of *U*. *lobata* extract, Fra. 4, (−)-trachelogenin (**1**) and clematoside-S (**2**).

Microorganisms Strain	Inhibition Zone (mm)					
	Extract ^a^	Fra. 4 ^b^	1 ^c^	2 ^c^	Negative Control ^d^	Positive Control ^e^
**Gram negative bacteria**						
*Eschericha coli* ATCC 25922	0	NT ^f^	0	0	0	35
*Salmonella typhimurium* ATCC 14028	0	NT	0	0	0	28
**Gram positive bacteria**						
*Staphylpcocuus aureus* ATCC 25923	0	NT	0	0	0	28
*Bacillus subtilis* ATCC 21216	0	NT	0	0	0	18
*Bacillus cereu*s ATCC 10231	0	NT	0	0	0	15
*Bacillus laterosporus* ATCC 64	0	NT	0	0	0	21
**Fungi**						
*Aspergillus flavus* ATCC 204304	0	NT	0	0	0	25
*Aspergillus niger* ATCC 16404	9	NT	0	9	0	20
*Rhizopus oryzae* ATCC 9363	0	NT	0	0	0	28
*Pencicillum citrinum* ATCC 14994	0	NT	0	0	0	26
**Yeasts ^g^**						
*Candida albicans* ATCC 50013	0	NT	0	0	0	0
*Saccharomyces cerevisiae* ATCC 204508	15	16	0	20	0	21
*Saccharomyces cerevisiae* AY529515.1	17	NT	0	11	0	21
*Saccharomyces cerevisiae* AJ746340.1	14	NT	0	12	0	21
*Saccharomyces cerevisiae* JX103178.1	14	NT	0	12	0	21
*Saccharomyces boulardii* KG254081.1	17	NT	0	13	0	21

^a^ The concentration of the 80% ethanol extract from *U*. *lobata* leaves was 2 mg/disk; ^b^ Fra. 4 (fraction 4) was obtained due to Scheme 1; ^c^ The concentration of compounds **1**–**2** was 2 mg/disk; ^d^ 95% methanol as the negative control; ^e^ Penicillin (2 mg/disk) as the positive control against bacteria and streptomycin (2 mg/disk) as the positive control against fungi and yeasts; ^f^ Not tested; ^g^ *Saccharomyces cerevisiae*\AY529515.1, *Saccharomyces cerevisiae*\AJ746340.1, *Saccharomyces cerevisiae*\JX103178.1, and *Saccharomyces boulardii*\KG254081.1 were isolated from spoiled grapes.

**Table 3:** MIC ^a^ and MBC ^b^ of clematoside-S (2) for different yeast strains.

Yest Strains	MIC (μg/mL)	MBC (μg/mL)
*Saccharomyces cerevisiae* ATCC 204505	0.61	2.42
*Saccharomyces cerevisiae*\AY529515.1	1.21	4.84
*Saccharomyces cerevisiae*\AJ746340.1	9.80	9.80
*Saccharomyces cerevisiae*\JX103178.1	1.21	4.84
*Saccharomyces boulardii*\KG254081.1	2.42	9.80

^a^ Minimum inhibitory concentration (MIC); ^b^ Minimum bactericidal concentration (MBC).

## 3. Experimental

### 3.1. General Procedure

ESIMS spectra were acquired on a Thermo Finnigan TSQ Quantum Ultra AM mass spectrometer system with an electrospray source operating in both positive and negative ion modes (Thermo Electron, San Jose, CA, USA). Fast atom bombardment (FAB) MS was obtained with a Jeol JMS-SX102A instrument (JEOL, Tokyo, Japan). Optical rotation values were measured on JASCO P-1020 polarimeter (JASCO, Tokyo, Japan). NMR spectra were recorded with a Bruker AV II-600 instrument (^1^H, 600 MHz; ^13^C, 125 MHz) (Bruker Co., Karlsruhe, Germany). Column chromatography was performed with MCI gel 100A (75–150 μm, Sci-Bio-Chem Co., Ltd., Chengdu, China). The HPLC system (Agilent 1200 Series Purification System, Aglilent, Santa Clara, CA, USA) consisted of an injector (G1328B), a column oven (35 °C), a pump (G1311A), a diode array detector (G1315D), and an Inertsil PREP-ODS column (6 × 250 mm i.d. with a particle size of 5 μm, GL-Science, Tokyo, Japan). The solvents used were all of HPLC-grade for HPLC analysis.

### 3.2. Plant Material and Regents

The leaves of *U*. *lobata* used in this study were purchased from Chengdu Medicinal Materials (Chengdu, China) and properly identified at the Department of Pharmacology, Hua Xi Medicinal Center of Sichuan University, China. A voucher specimen is deposited in the Rice Research Institute, Sichuan Agricultural University (No. 20090603). Penicillin and streptomycin were obtained from Sichuan Changwei Pharmaceutical Co., Ltd. (Chengdu, China). Agar, beef extract, sucrose and peptone were purchased from Chengdu Best Reagents Co., Ltd. (Chengdu, China). All other reagents used were of analytical grade.

### 3.3. Extraction and Isolation of the Active Compound

The dried leaves of *U*. *lobata* (100 g) was crushed into powders with a mixer, followed by extraction with 80% ethanol (2000 mL) in total for 3 day at room temperature and filtered. Evaporation of the solvent under reduced pressure yielded the 80% ethanol extract (15.3 g), which was applied on a MCI gel column (3.5 × 20 cm) with a water-methanol gradient to give four fractions (fraction 1, water (800 mL, ~6.4 g); fraction 2, 50% methanol in water (800 mL, 0.7 g); fraction 3, 70% methanol in water (800 mL, 2.1 g); fraction 4, 100% methanol (600 mL, 0.8 g)). Consequently, fraction 4 was further purified by preparative HPLC (column, Inertsil ODS 6 × 250 mm i.d. with a particle size of 5 μm, GL-Science; mobile phase, water-methanol = 20:80; flow rate, 1 mL/min; detection, UV 220 nm) to yield 24 mg of (−)-trachelogenin (**1**, *t*_R_ 12.8 min) and 85 mg of clematoside S (**2**, *t*_R_ 15.4 min).

### 3.4. Acid Hydrolysis and GC-MS of Sugars

A part (2.1 mg) of **2** was dissolved in 2 M HCl (5% MeOH, 1 mL) and heated at 95 °C for 90 min. After the hydrolysis, EtOAc (2 mL) was added to the solution and voltexed to remove aglycone. This extraction was repeated twice. The aqueous residue was divided into two microtubes, and subjected to a centrifuge-concentration *in vacuo*. The dried concentrate was converted to *N*-methoximes in 50 μL of pyridine containing methoxyamine hydrochloride (15 mg/mL) overnight at room temperature. To this reaction mixture, 30 mL of MSTFA (*N*-methyl-*N*-trimethylsilyltrifuloroacetamide) was added and heated at 45 °C for 20 min for trimethylsilylation. The trimethylsilylated *N*-methoxime sugars were submitted to GC-MS analysis under the following condition: column, DB-1 (0.25 mm i.d. × 25 m, 0.25 μm); injector temperature, 250 °C; carrier gas, He (0.9 mL/min); oven temperature program, 70 °C (1 min), 70–150 °C (20 °C/min), 150–180 °C (2 °C/min); ionization, EI (70 eV).

### 3.5. Antimicrobial Activity

The tested microorganisms contained Gram-negative bacteria (*Escherichia coli* ATCC 25922, *Salmonella typhimurium* ATCC 14028), Gram-positive bacteria (*Staphylpcoccus aureus* ATCC 25923, *Bacillus subtilis* ATCC 21216, *Bacillus cereus* ATCC 10231, *Bacillus laterosporus* ATCC 64), fungi (*Aspergillus niger* ATCC 16404, *Aspergillus flavus* ATCC 204304, *Pencicillium citrinum* ATCC 14994, and *Rhizopus oryzae* ATCC 9363) and yeasts (*Candida albicans* ATCC 50013, *Saccharomyces cerevisiae* ATCC 204508, *Saccharomyces cerevisiae*\AY529515.1, *Saccharomyces cerevisiae*\AJ746340.1, *Saccharomyces cerevisiae*\JX103178.1, and *Saccharomyces boulardii*\KG254081.1), and were obtained from the Key Laboratory of Food Science and Technology of Sichuan Province, Sichuan University. The yeasts (*Saccharomyces cerevisiae*\AY529515.1, *Saccharomyces cerevisiae*\AJ746340.1, *Saccharomyces cerevisiae*\JX103178.1, and *Saccharomyces boulardii*\KG254081.1) were isolated from spoiled grapes and identified by morphology, biochemical tests and ITS sequence analysis. All the microorganisms were maintained on nutrient agar at 4 °C and were sub-cultured every month in our laboratory. In the present test, nutrient agar culture medium was for bacteria and the medium of potatoes was for fungi and yeasts strains.

Antimicrobial activity of the test sample was determined by Oxford plate method [24]. In short, bacterial cultures were diluted to obtain a bacterial suspension of 10^6^ CFU/mL with sterile water. Petri plates containing 20 mL of nutrient agar were inoculated with 0.2 mL of bacterial culture and were allowed to dry in sterile chamber. The Oxford plates (6 mm in diameter) were impregnated with 0.1 mL of test sample in 95% methanol and placed on the inoculated agar. Penicillin and streptomycin were used as the positive control for bacteria and fungus, respectively. The inoculated plates of bacteria were incubated at 37 °C for 24 h, and fungi were incubated at 35–37 °C for 48 h. The antimicrobial activity was evaluated by measuring the zone of inhibition against the test organisms.

The minimum inhibitory concentration (MIC) of the test sample was evaluated for the yeast strains which were determined by the method of broth dilution [25]. An aliquot of 2 mL of the medium of potatoes was placed into each tube, and all tubes were autoclaved at 121 °C. Test sample (filtered, 0.22 μm) was added to the tubes to keep the final concentrations ranging from 0.06 to 40 μg/mL. The test yeast suspension was added into to the inoculum size of 10^6^ CFU/mL. Then, the inoculated tubes were incubated at 37 °C for 18–24 h. The MIC was evaluated by measuring the turbidity of inoculated culture media. Another liquid medium without adding any yeast was prepared as the negative control. The minimum inhibitory concentration at which no microorganism grew in the culture media was defined as the value of MIC. The minimum bactericidal concentration (MBC) of the test sample was determined according to the MIC values. The sample showing no increases in turbidity were streaked on potatoes medium and incubated at 37 °C for 18–24 h. The lowest concentration of the test sample where was no viable yeasts was identified as the value of MBC.

## 4. Conclusions

In the present study, (−)-trachelogenin (**1**) and clematoside-S (**2**) were isolated from the leaves of *U*. *lobata* for the first time by a combination of chromatographic methods and their chemical structure was identified by MS and extensive NMR data analysis. Furthermore, to the best of our knowledge, this is the first study demonstrating the antimicrobial activity of clematoside-S. In addition, clematoside-S shows a specific anti-yeast activity against *S*. *cerevisiae* strains, which are one of the most significant spoilage yeasts for juice and soft drinks.

## References

[1] Goretti M., Turchetti B., Buratta M., Branda E., Cxorazzi L., Vaughan-Martini A., Buzzini P. (2009). *In vitro* antimycotic activity of a *Williopsis saturnus* killer protein against food spoilage yeasts. Int. J. Food Microbiol..

[2] Belletti N., Kamdem S.S., Patrignani F., Lanciotti R., Covelli A., Gardini F. (2007). Antimicrobial activity of aroma compounds against *Saccharomyces cerevisiae* and improvement of microbiological stability of soft drinks as assessed by logistic regression. Appl. Environ. Microbiol..

[3] Loureiro V., Querol A. (1999). The prevalence and control of spoilage yeasts in foods and beverages. Trends Food Sci. Technol..

[4] Tserennadmid R., Takó M., Galgóczy L., Papp T., Pesti M., Vágvölgyi C., Almássy K., Krisch J. (2011). Anti yeast activities of some essential oils in growth medium, fruit juices and milk. Int. J. Food Microbiol..

[5] Burt S. (2004). Essential oils: Their antibacterial properties and potential applications in foods—A review. Int. J. Food Microbiol..

[6] Tyagi A.K., Gottardi D., Malik A., Guerzoni M.E. (2013). Anti-yeast activity of mentha oil and vapours through *in vitro* and *in vivo* (real fruit juices) assays. Food Chem..

[7] De las Heras B., Slowing K., Benedí J., Carretero E., Ortega T., Toledo C., Bermejo P., Iglesias I., Abad M.J., Gómez-Serranillos P. (1998). Antiinflammatory and antioxidant activity of plants used in traditional medicine in Ecuador. J. Ethnopharmacol..

[8] Pieme C.A., Penlap V.N., Ngogang J., Costache M. (2010). *In vitro* cytotoxicity and antioxidant activities of five medicinal plants of Malvaceae family from Cameroon. Environ. Toxicol. Pharmacol..

[9] Mazumder U.K., Gupta M., Manikandan L., Bhattacharya S. (2001). Antibacterial activity of *Urena lobata*root. Fitoterapia.

[10] Meléndez P.A., Capriles V.A. (2006). Antibacterial properties of tropical plants from Puerto Rico. Phytomedicine.

[11] Xie Z., Zeng H.S., Liu C., Chen Y., Zhong M.Y. (2013). Simultaneous quantification of protocatechuic acid, caffeic acid, quercetin and kaempferol in *Urena lobata* L. by HPLC-VWD. Lat. Am. J. Pharm..

[12] Morelli G.F., Cairoli P., Speranza G., Alamgir M., Rajia S. (2006). Triglycerides from *Urena lobata*. Fitoterapia.

[13] Jia L., Bi Y.F., Jing L.L., Zhou S.A., Kong D.Y. (2010). Two new compounds from *Urena lobata* L.. J. Asian Nat. Prod. Res..

[14] John L.M.D., Tinto W.F. (1992). Revised ^13^C-NMR assignments for the biologically active butyrolactone (−)-trachelogenin. J. Nat. Prod..

[15] Lima O.O.A., Braz-Filho R. (1997). Dibenzylbutyrolactone lignans and coumarins from *Ipomoea cairica*. J. Braz. Chem. Soc..

[16] Boldizsár I., Kraszni M., Tóth F., Noszál B., Molnár-Perl I. (2010). Complementary fragmentation pattern analysis by gas chromatography–mass spectrometry and liquid chromatography tandem mass spectrometry confirmed the precious lignan content of *Cirsium* weeds. J. Chromatogr. A.

[17] Yokosuka A., Sano T., Hashimoto K., Sakagami H., Mimaki Y. (2009). Triterpene glycosides from the whole plant of *Anemone hupehensis* var. japonica and their cytotoxic activity. Chem. Pharm. Bull..

[18] Mizutani K., Ohtani K., Wei J.X., Tanaka O. (1984). Saponins from *Anemone rivularis*. Planta Med..

[19] Sati O.P., Uniyal S.K., Bahuguna S., Kikuchi T. (1990). Clematoside-S, a triterpenoid saponin from the roots of *Clematis grata*. Phytochemistry.

[20] Hara S., Okabe H., Mihashi K. (1987). Gas-liquid chromatographic separation of aldose enantiomers as trimethylsilyl ethers of methyl 2-(polyhydroxyakyl)-thiazolidine-4-(*R*)-carboxylates. Chem. Pharm. Bull..

[21] Mitaine-Offer A.C., Pénez N., Miyamoto T., Delaude C., Mirjolet J.F., Duchamp O., Lacaille-Dubois M.A. (2010). Acylated triterpene saponins from the roots of *Securidaca longepedunculata.*. Phytochemistry.

[22] Pawelec S., Jedrejek D., Kowalczyk M., Pecio L., Masullo M., Piacente S., Macias F.A., Simonet A.M., Oleszek W., Stochmal A. (2013). Triterpene saponins from the aerial parts of *Trifolium medium* L. var. *sarosiense*. J. Agric. Food Chem..

[23] Yan L.H., Xu L.Z., Lin L., Yang S.L., Feng Y.L. (2009). Triterpenoid saponins from the stems of *Clematis parviloba*. J. Asian Nat. Prod. Res..

[24] Zeng W.C., Jia L.R., Zhang Y., Cen J.Q., Chen X., Gao H., Huang Y.N. (2011). Antibrowning and antimicrobial activities of the water-soluble extract from pine needles of *Cedrus deodara*. J. Food Sci..

[25] Zeng W.C., Zhu R.X., Jia L.R., Gao H., Zheng Y., Sun Q. (2011). Chemical composition, antimicrobial and antioxidant activities of essential oil from *Gnaphlium affine*. Food Chem. Toxicol..

